# Equity and efficiency of health care resource allocation in Jiangsu Province, China

**DOI:** 10.1186/s12939-020-01320-2

**Published:** 2020-11-27

**Authors:** Qian Li, Jianjun Wei, Fengchang Jiang, Guixiang Zhou, Rilei Jiang, Meijuan Chen, Xu Zhang, Wanjin Hu

**Affiliations:** 1 Institute of Literature in Chinese Medicine, Nanjing University of Chinese Medicine, Nanjing, 210023 Jiangsu P.R. China; 2 Shanghai First Maternity and Infant Hospital, Tongji University School of Medicine, 200040 Shanghai, P.R. China; 3 Department of Construction Management of Real Estate, School of Economics and Management, Tongji University, Shanghai, 200092 P.R. China; 4 Shanghai Shenkang Hospital Development Centre, Shanghai, 200092 P.R. China; 5 Taizhou Polytechnic College, Taizhou, 225300 Jiangsu P.R. China; 6 School of Basic Medicine Science, Shanghai University of Traditional Chinese Medicine, Shanghai, 200032 P.R. China; 7 School of Medicine & Holistic Integrative medicine, Nanjing University of Chinese Medicine, Jiangsu Nanjing, 210023 P.R. China; 8 Nanjing Municipal Government, Jiangsu Nanjing, 210008 P.R. China

**Keywords:** Health care resource, Equity, Efficiency, Productivity

## Abstract

**Background:**

Jiangsu was one of the first four pilot provinces to engage in comprehensive health care reform in China, which has been on-going for the past 5 years. This study aims to evaluate the equity, efficiency and productivity of health care resource allocation in Jiangsu Province using the most recent data, analyse the causes of deficiencies, and discuss measures to solve these problems.

**Methods:**

Data were extracted from the Jiangsu Health/Family Planning Statistical Yearbook (2015–2019) and Jiangsu Statistical Yearbook (2015–2019). The Gini coefficient (G), Theil index (T) and health resource density index (HRDI) were chosen to study the fairness of health resource allocation in Jiangsu Province. Data envelopment analysis (DEA) and the Malmquist productivity index (MPI) were used to analyse the efficiency and productivity of this allocation.

**Results:**

From 2014 to 2018, the total amount of health resources in Jiangsu Province increased. The G of primary resource allocation by population remained below 0.15, and that by geographical area was between 0.14 and 0.28; additionally, the G of health financial resources was below 0.26, and that by geographical area was above 0.39. T was consistent with the results for G and Lorenz curves. The HRDI shows that the allocated amounts of health care resources were the highest in southern Jiangsu, except for the number of health institutions. The average value of TE was above 0.93, and the DEA results were invalid for only two cities. From 2014 to 2018, the mean TFPC in Jiangsu was less than 1, and the values exceeded 1 for only five cities.

**Conclusion:**

The equity of basic medical resources was better than that of financial resources, and the equity of geographical allocation was better than that of population allocation. The overall efficiency of health care resource allocation was high; however, the total factor productivity of the whole province has declined due to technological regression. Jiangsu Province needs to further optimize the allocation and increase the utilization efficiency of health care resources.

**Supplementary Information:**

The online version contains supplementary material available at 10.1186/s12939-020-01320-2.

## Background

Health care resources are the basis for the development of health services, and the rationality of their allocation not only affects residents’ health level but also plays an important role in the sustainable development of medical and health services [1, 2]. Therefore, research on the allocation of health resources has long been a top priority [3]. Health resource allocation is not a simple spatial distribution, and more attention should be paid to whether the structure of health resource allocation is reasonable and fair. Additionally, this allocation should be optimized as much as possible to maximize efficiency and quality [4, 5].

In recent years, due to economic development, an ageing population, the prevalence of chronic diseases and the improvement in health concepts, higher standards have been established for the allocation of health resources. The equity and efficiency of health resource allocation and health service utilization are attracting increasing attention in China [6, 7]. To establish a “safe, convenient, cheap and effective” medical service system, China has actively launched new medical reforms since 2009. The Chinese government has constantly adjusted the medical system to improve the equity and efficiency of health resources and services. In the past decade, the Chinese government has continuously increased medical investment, established universal health insurance coverage and gradually improved the population’s health level [8, 9]. However, many studies have shown that due to the different economic development levels in eastern, central and western China, as well as the imbalance of urban and rural development, an unfair distribution of medical resources still exists, and the efficiency of medical resources in each province is generally low; thus, this situation needs to be improved [10, 11]. To further deepen the medical reform, China has set up reform pilot programmes in several provinces and cities.

Jiangsu was one of the first four pilot provinces to engage in comprehensive health care reform in China. Jiangsu is located in the eastern coastal area of China and has 13 prefecture-level cities, such as Nanjing, Zhenjiang, Suzhou and Huaian [12]. Jiangsu Province is the fifth most populous and the most densely populated of the 22 provinces of China. The GDP of Jiangsu Province in 2018 was 9259.5 billion yuan, ranking second in China, and its per capita GDP reached 115,168.4 yuan, ranking first in China. These data show that the economy of Jiangsu Province is equivalent to those of “middle and upper class” developed countries. To use these achievements in economic development to meet people’s growing health needs, Jiangsu Province has issued many medical reform policies in recent years [8].

Jiangsu Province has implemented comprehensive health care reform for the past 5 years, and the year 2020 is also the last year of the Healthy China 2020 strategy [13]. This study aims to evaluate the equity, efficiency and productivity of health care resource allocation in Jiangsu Province using the most recent data; analyse the causes of deficiencies; discuss measures to solve these problems; and provide suggestions for the sustainable development of medical treatment in Jiangsu Province as well as provide a reference for the further advancement of medical reform and implementing the Healthy China 2030 strategy.

## Methods

### Data sources

Data were extracted from the Jiangsu Health/Family Planning Statistical Yearbook (2015–2019) and Jiangsu Statistical Yearbook (2015–2019), which cover 13 cities in Jiangsu Province. The series of longitudinal data were used to analyse the equity and efficiency trends in Jiangsu from 2014 to 2018.

### Setting

On the basis of the economic development level, geographical position and the Jiangsu Statistical Yearbook, the 13 cities in Jiangsu Province were divided into three groups: the most economically developed southern zone (including Nanjing, Zhenjiang, Suzhou, Wuxi and Changzhou), the moderately developed middle zone (including Yangzhou, Taizhou and Nantong) and the less economically active northern zone (including Xuzhou, Lianyungang, Suqian, Huaian, and Yancheng). Health care institutions included hospitals, primary health institutions, professional public health institutions and other health institutions.

### Indicators and measurement tools

Considering representation, stability, availability and independence, capital, labour and financial investment are deemed to be important input variables in the delivery of health services. The number of institutions and beds represents the capital, the number of health workers represents labour, and the government financial subsidy and total expenditure represent financial investment. Health workers include physicians, registered nurses, assistant physicians, medical personnel, other technical personnel and health administrative staff. They were chosen as input indicators to measure equity. The annual numbers of outpatient visits, the hospitalization rate and general income represent output indicators.

#### Gini coefficients and Lorenz curves

The Gini coefficient is the optimal tool to assess the equity of health resource allocation in terms of demographic and geographical aspects [14]. The Gini coefficient is derived from the Lorenz curve. With regard to the Lorenz curve, the x-axis represents the cumulative percentage of population or geography, and the y-axis represents the cumulative percentage of health care resources (institutions, beds, health workers, government financial subsidy income and total expenditure). A 45° line indicates absolute equity. A larger distance from the diagonal line indicates greater unfairness. Formula (1) is used to calculate the Gini coefficients.

1 $$ G=1-{\sum}_{i=0}^{k-1}\left({Y}_i+1+{Y}_i\right)\left({X}_i+1-{X}_i\right), $$

*X*_*i*_: cumulative percentage of health resources in the *ith* city of Jiangsu Province after ranking according to the per capita or regional average share of health resources.

*Y*_*i*_: cumulative percentage of population or geography in the *ith* city of Jiangsu Province after ranking according to the per capita or regional average share of health resources.

*k*: total number of cities.

*G*: value of the Gini coefficient.

#### Theil index

The Theil index is used to analyse the inequity of resource allocation. The advantage of the Theil index is that it can measure the contributions of intra- and inter-regional differences to total inequity [15]. Formula (2) is used to calculate the total Theil index.

2 $$ T={\sum}_{i=1}^n{P}_i\mathit{\log}\frac{P_i}{E_i}, $$

*P*_*i*_: percentage of population or geography of each city in Jiangsu Province.

*E*_*i*_: percentage of health resources of each city in Jiangsu Province.

*T*: value of the total Theil index.

The total Theil index can be decomposed into the intra-Theil index and inter-Theil index [16]. Formula (3) is used to calculate the intra-Theil index. Formula (4) is used to calculate the inter-Theil index.

$$ T={T}_{intra}+{T}_{inter} $$

3 $$ {T}_{intra}={\sum}_{g=1}^k{P}_g{T}_g, $$

4 $$ {T}_{inter}={\sum}_{g=1}^k{P}_g\mathit{\log}\frac{P_g}{E_g}, $$

*T*_*intra*_: value of the intra-Theil index. In this study, it represents the intra-different distribution of health resources in southern, central and northern areas of Jiangsu Province.

*T*_*inter*_: value of the inter-Theil index. In this study, it represents the inter-different allocation of health resources among southern, central and northern areas of Jiangsu Province.

*P*_*g*_: proportion of population or geography of three regions (southern, central and northern areas) for the overall number of Jiangsu Province.

*E*_*g*_: proportion of health resources of three regions (southern, central and northern areas) for the overall number of Jiangsu Province.

*T*_*g*_: value of the Theil index of three regions (southern, central and northern areas).

The contribution rates of *T*_*intra*_ and *T*_*inter*_ can be calculated by dividing the total Theil index [17].

#### Health resource density index (HRDI)

The HRDI displays the influence of population and geographical factors on the agglomeration of health resources while avoiding bias caused by a single population or geographical aspect. Formula (5) is used to calculate the health resource density index (HRDI).

5 $$ HRDI=\frac{HR_i}{\sqrt{A_i{P}_i}}, $$

*HR*_*i*_: health resource quantity of the *ith* region.

*A*_*i*_: geography of the *ith* region.

*P*_*i*_: population of the *ith* region.

*HRDI*: value of the Health Resource Density Index.

#### Data envelopment analysis (DEA) model

DEA is a nonparametric method that evaluates the performance of mathematical planning models, including the relative efficiency of decision-making units (DMUs), using multiple input and output indicators [18, 19]. The DEA model comprises the CCR model (the Charnes, Cooper, and Rhodes model) and the BCC model (the Banker, Charnes, and Cooper model) [20, 21]. A careful examination of the actual situation, including imperfect competition, financial constraints and government regulations, reveals that health institutions in China always run at a suboptimal scale [20]. Thus, we selected the BCC model to analyse the research data.

In the BCC model, technical efficiency (TE) can be disassembled into pure technical efficiency (PTE) and scale efficiency (SE). Formula (6) is used to calculate the technical efficiency (TE).

6 $$ TE= PTE\times SE, $$

TE expresses the production efficiency of DMU, which is based on input resource. PTE reflects the advance in productivity resulting from the efforts of managers and workers and the effective combination of production factors. SE indicates different levels of changes in DMU’s economies of scale [11]. Values of TE, PTE and SE of 1 indicate efficiency.

#### Total factor productivity change (TFPC)

Whereas DEA measures relative efficiency for a period of time, TFPC measures dynamic changes in productivity from time t to time t + 1 [11]. TFPC can be decomposed into technical efficiency change (TEC) and technological change (TC); TEC can be further divided into pure technical efficiency change (PTEC) and scale efficiency change (SEC) [22]. Formula (7) is used to calculate the total factor productivity change (TFPC). Formula (8) is used to calculate the technical efficiency change (TEC).

7 $$ TFPC= TEC\times TC, $$

8 $$ TEC= PTEC\times SEC, $$

Values of TFPC, TEC, TC, PTEC and SEC above 1 indicate improvement. The DEA and TFPC were calculated using DEAP V.2.1.

## Results

### Current situation of health resource allocation in Jiangsu Province

#### Primary health resources

**Fig. 1:**
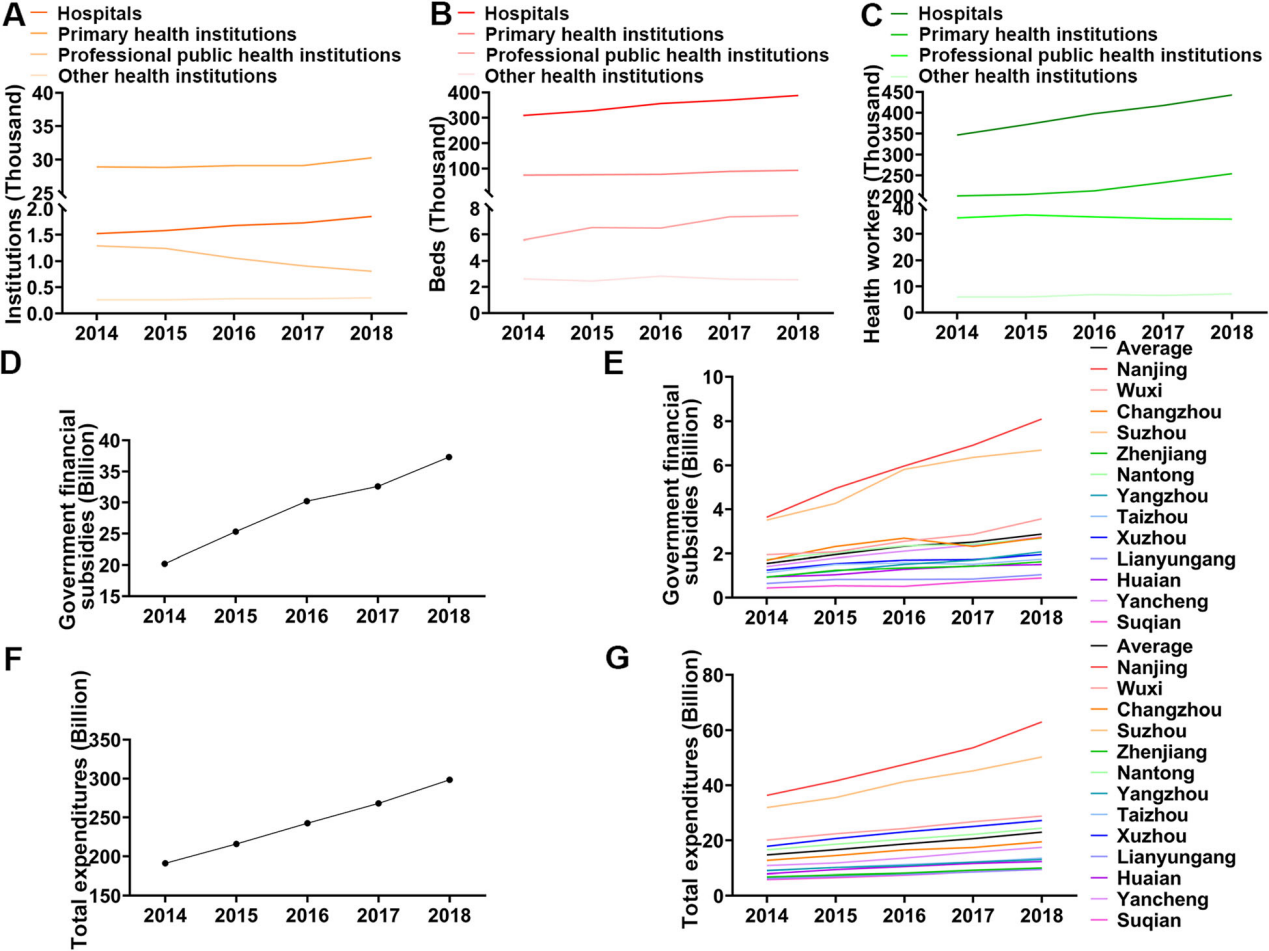
The allocation of health resources in Jiangsu Province from 2014 to 2018. **a-c** show the number of different kinds of institutions, beds and health workers. **d, f** denote the government financial subsidies and total expenditures in Jiangsu Province. **e, g** denote the distribution of government financial subsidies and total expenditures in 13 cities of Jiangsu Province

From 2014 to 2018, the total amount of primary health resources increased in Jiangsu Province. In a general sense, the primary resources include capital and labour [11]. In terms of capital, there was a small fluctuation in the number of health institutions from approximately 32,000 before 2017 to 33,253 in 2018 (Table 1). The total number of beds in the whole province has maintained stable growth, reaching 491,522 by 2018. The primary resources per thousand persons and per square kilometre all increased from 2014 to 2018 (Table 1). Regarding labour, the total number of stable health workers exhibited a similar growth trend to that found for beds, reaching 739,294 by 2018. The distribution of various health institutions shows that the number of all kinds of hospitals and primary health institutions grew stably, while the number of professional public health institutions dropped rapidly (Fig. 1a). The majority of beds and health workers are found in hospitals, so the total numbers grew as hospitals grew (Fig. 1b, c).

**Table 1:** Primary resource allocation in Jiangsu Province from 2014 to 2018

Year	Institutions			Beds			Health workers		
	/1000 persons	/km^2^	Total	/1000 persons	/km^2^	Total	/1000 persons	/km^2^	Total
2014	0.4020	0.2985	32,000	4.9283	3.6594	392,293	7.4070	5.5000	589,598
2015	0.4003	0.2978	31,925	5.1857	3.8583	413,612	7.7601	5.7737	618,945
2016	0.4017	0.2998	32,135	5.5394	4.1334	443,100	8.1786	6.1027	654,210
2017	0.3990	0.2989	32,037	5.8514	4.3825	469,805	8.6286	6.4626	692,794
2018	0.4130	0.3102	33,253	6.1051	4.5851	491,522	9.1826	6.8964	739,294

#### Financial resources

For financial resources, we counted government financial subsidies and total expenditures. From 2014 to 2018, government financial subsidies continued to grow to 37.3 billion yuan (Fig. 1d). The number of subsidies was 83.5% greater in 2018 than in 2014. At the city level, government financial subsidies in Nanjing and Suzhou were significantly higher than those in other cities in the province and the provincial average (Fig. 1e). The total expenditure also shows a trend of rapid growth (Fig. 1f). Each city in Jiangsu Province grew its total expenditure in the past 4 years, especially Nanjing and Suzhou (Fig. 1g). Suqian, Lianyungang and Zhenjiang are the three cities with the lowest total expenditure (less than 10 billion by 2018), and the growth rate is relatively slow. The government financial subsidy mainly comes from the tax revenue of each city. The financial subsidy income of Nanjing, Suzhou and other cities in the most economically developed southern zone is significantly higher than Suqian, Lianyungang and other cities in the less economically active northern zone. The total expenditures are closely related to the total population (including permanent residents and floating population) of each city, which directly reflects people’s medical needs. Therefore, the level and growth rate of medical financial resources in each city are related mainly to the economic development of each city and the local medical demand.

### The equity of health resource allocation in Jiangsu Province

#### Primary health resources

**Fig. 2:**
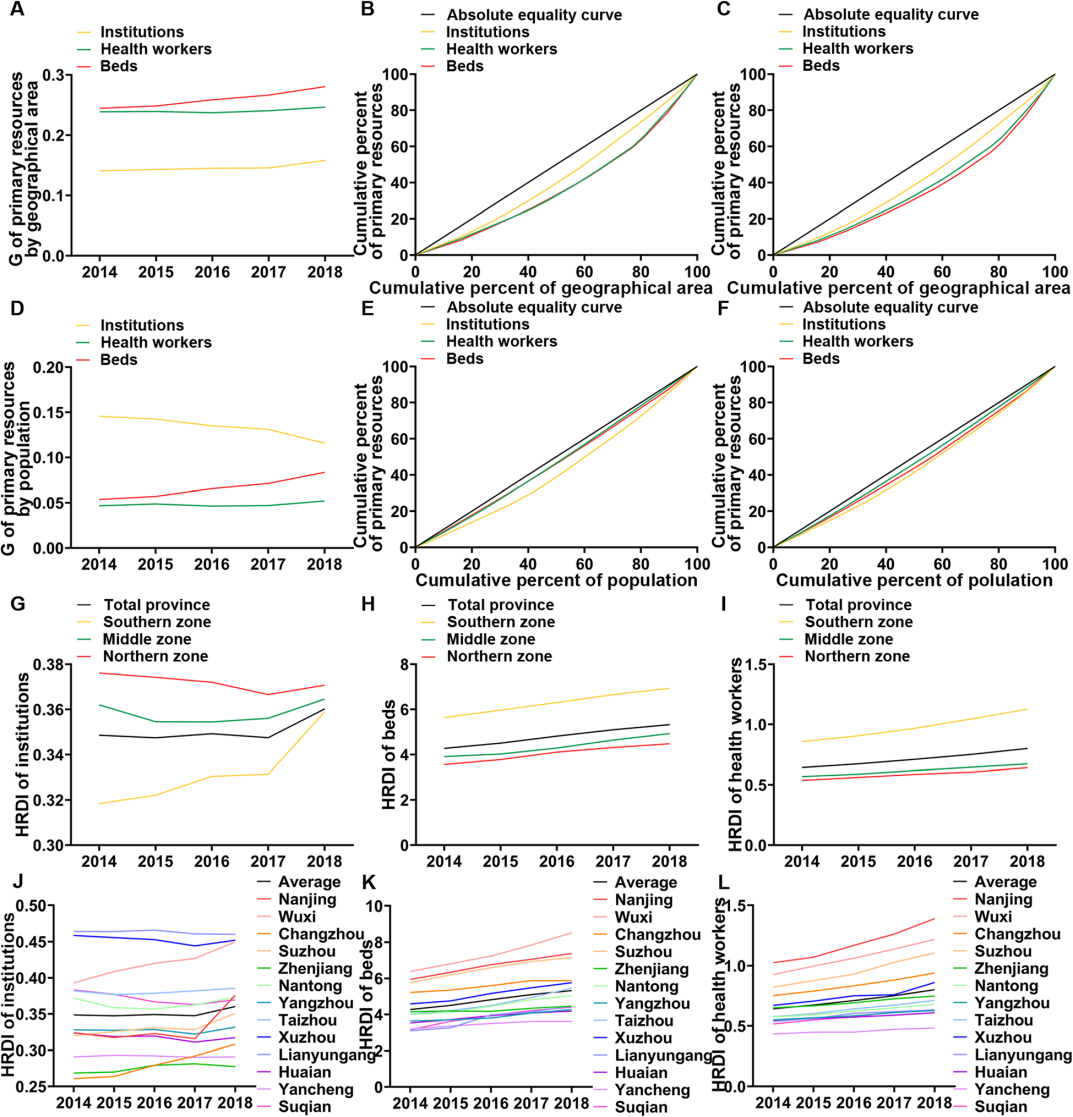
The equity of primary health resource allocation in Jiangsu Province from 2014 to 2018. **a** shows the G of primary resources allocated by geographical area. **b, c** show the Lodz curves of primary resources allocated by geographical area in 2014 and 2018, respectively. **d** shows the G of primary resources allocated by population. **e, f** show the Lorenz curves of primary resources allocated by population in 2014 and 2018, respectively. **g-i** denote the HRDI of institutions, beds and health workers in different regions. **j-l** denote the HRDI of institutions, beds and health workers in 13 cities

With the consideration of demographic and geographical dimensions, we analysed the two aspects of equity. The Gini coefficients of primary resource allocation by population remained below 0.15, indicating fairness (Fig. 2d). However, for primary resource allocation by geographical area, the G of beds and health workers remained above 0.2 and tended to grow, indicating lower fairness (Fig. 2a). The overall performance of the Lorenz curves was consistent with the Gini coefficients (Fig. 2b, c, e, f and S1A-F). The curves of institutions were closer to the absolute equality curve compared to those of health workers and beds by geographical area, which may be due to the outflow of talent in less economically active areas (Fig. 2b, c and S1A-C).

The Theil index was used to analyse the inequity of health resource allocation, and the results were consistent with the results of the Gini coefficient and the Lorenz curves (Table 2). Further analysis of the perspective of intragroup and intergroup contribution rates shows that the intraregional contribution rate of institutions is the highest; however, the interregional contribution rate is highest for beds and health workers. Subsequently, we continued to decompose intraregional differences (Table 3), which shows that the inequality in primary health resource allocation mostly came from northern Jiangsu Province. The contribution rate of internal differences in northern Jiangsu to the allocation of institutions was more than 75%, and the contribution rate to the allocation of beds and health workers was more than 64 and 71%, respectively. However, the differences in northern Jiangsu decreased, whereas those in southern and central Jiangsu increased. To clarify the equity of primary resource allocation in different regions of Jiangsu Province, we calculated the T of every region in Table 4. The T of institutions, beds and health workers was the highest in northern Jiangsu and the lowest in central Jiangsu, which means that the equity of primary health resource allocation was the worst in northern Jiangsu and the best in central Jiangsu. It is worth noting that the T of the institutions, beds and health workers in southern Jiangsu is increasing annually.

**Table 2:** T of primary health resources allocation in Jiangsu Province

Year	Theil index			Contribution rate of intra-region (%)			Contribution rate of inter-region (%)		
	Institutions	Beds	Health workers	Institutions	Beds	Health workers	Institutions	Beds	Health workers
2014	0.0141	0.0388	0.0409	86.74	23.55	24.64	13.26	76.45	75.36
2015	0.0144	0.0390	0.0423	85.12	23.40	25.24	14.88	76.60	74.76
2016	0.0149	0.0383	0.0460	81.46	27.18	27.33	18.54	72.82	72.67
2017	0.0152	0.0393	0.0491	79.41	28.79	24.50	20.59	71.21	79.41
2018	0.0176	0.0414	0.0541	71.96	31.83	28.14	28.04	68.17	71.96

**Table 3:** Proportion of differences in contribution in the south, middle and north

Year	Institutions			Beds			Health workers		
	South	Middle	North	South	Middle	North	South	Middle	North
2014	18.97	2.87	78.16	25.02	2.45	72.53	25.51	1.08	73.42
2015	20.58	2.57	76.84	30.03	3.13	66.84	25.86	1.19	72.94
2016	19.61	2.66	77.73	31.14	4.02	64.84	24.81	1.58	73.61
2017	19.31	3.30	77.39	30.79	4.27	64.94	28.03	1.68	70.29
2018	21.86	2.57	75.57	30.14	4.97	64.90	25.51	1.73	72.76

**Table 4:** T of primary health resources allocation in the south, middle, north

Year	Institutions			Beds			Health workers		
	South	Middle	North	South	Middle	North	South	Middle	North
2014	0.0088	0.0016	0.0185	0.0086	0.0010	0.0128	0.0097	0.0005	0.0143
2015	0.0095	0.0015	0.0182	0.0103	0.0013	0.0118	0.0104	0.0006	0.0150
2016	0.0090	0.0015	0.0182	0.0122	0.0019	0.0130	0.0118	0.0009	0.0179
2017	0.0088	0.0018	0.0180	0.0131	0.0022	0.0142	0.0127	0.0009	0.0163
2018	0.0105	0.0015	0.0185	0.0150	0.0030	0.0165	0.0146	0.0012	0.0214

The HRDI analysis provides an intuitive perspective. The HRDI of beds and health workers in different regions of Jiangsu Province has gradually increased (Fig. 2h, i). The HRDI of institutions was the highest in northern Jiangsu (Fig. 2g), while that of beds and health workers was highest in southern Jiangsu. However, the difference between the HRDIs of institutions in southern Jiangsu, northern Jiangsu and central Jiangsu decreased. The results of a further analysis of 13 cities are consistent with these observations (Fig. 2j-l). The HRDI of primary medical resources in Jiangsu is on the rise, but there are still differences among regions in the province.

#### Financial resources

**Fig. 3:**
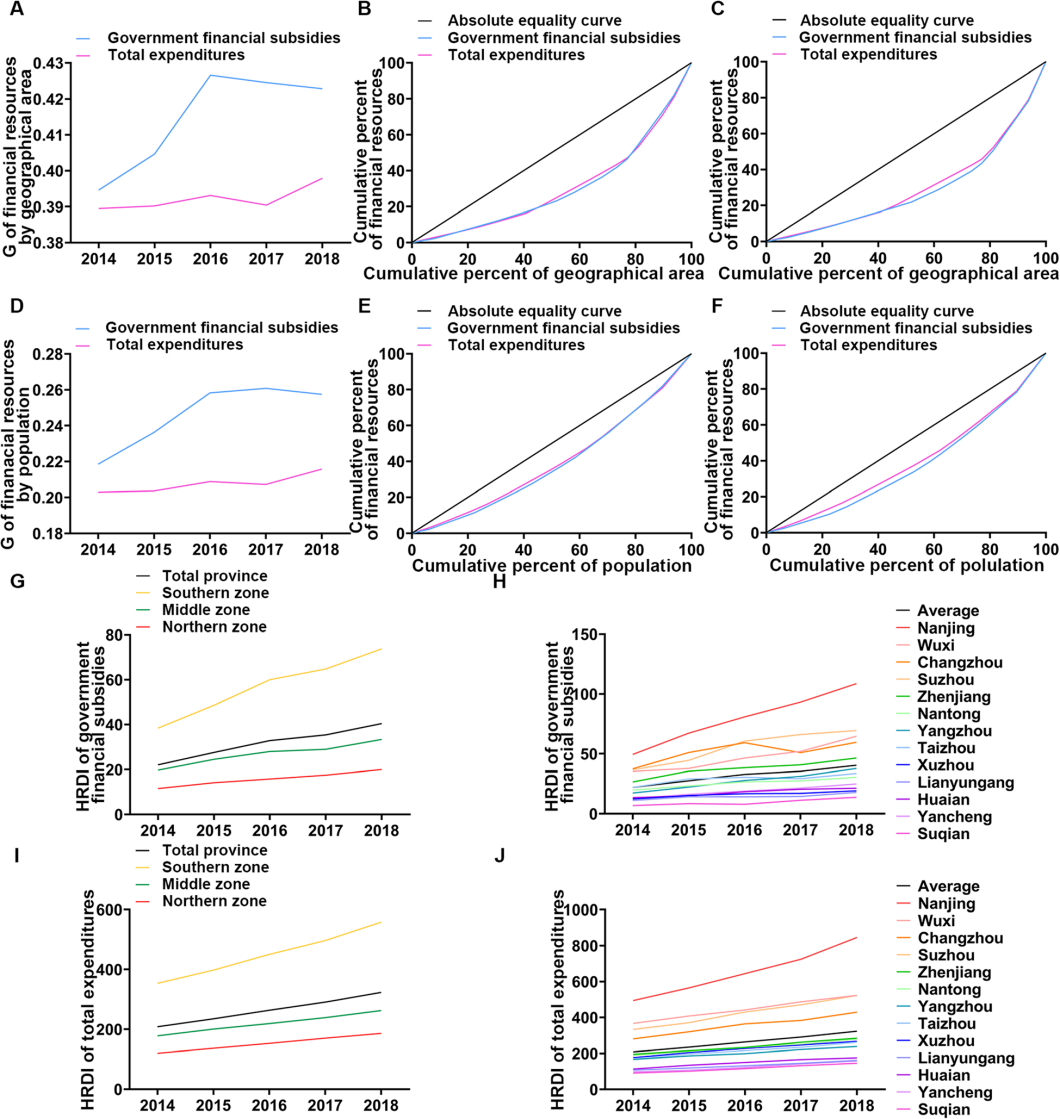
The equity of financial resource allocation in Jiangsu Province from 2014 to 2018. **a** shows the G of financial resources allocated by geographical area. **b, c** show the Lorenz curves of financial resources allocated by geographical area in 2014 and 2018, respectively. **d** shows the G of financial resources allocated by population. **e, f** show the Lorenz curves of financial resources allocated by population in 2014 and 2018, respectively. **g, i** denote the HRDI of government financial subsidies and total expenditures in different regions. **h, j** denote the HRDI of government financial subsidies and total expenditures in 13 cities

In 2018, the G values of health financial subsidies and total expenditures in Jiangsu Province were 0.423 and 0.398, respectively, which were higher than those in 2014 (Fig. 3a). This finding indicates that the allocation of health financial resources by geographical distribution in Jiangsu Province is relatively unfair. However, the G of health financial resources allocated by population is between 0.2 and 0.3 (Fig. 3d), which is lower than that for resources allocated by geographical location, indicating that the allocation is relatively fair. The overall performance of the Lorenz curves was consistent with the Gini coefficients (Fig. 3b, c, e, f and S1A-F); the curve of health financial subsidies and total expenditures of the population distribution is closer to the curve of absolute equality, and the equity of total expenditures is higher than that of financial subsidies.

Further analysis with T showed that its equity was similar to G and that the inequality was due mainly to inter-regional differences. The contribution rates of intraregional financial subsidies and total expenditure were approximately 92 and 82%, respectively (Table 5). Further study of the subregion shows that the contribution rate of central Jiangsu to the difference in financial subsidy income and total expenditure is the lowest. The contribution rate of northern Jiangsu is decreasing, while that of southern Jiangsu is increasing (Table 6). To clarify the equity of financial resource allocation in different regions of Jiangsu Province, we calculated the T for every region (Table 7). The T of financial subsidy income and total expenditure was the highest in southern Jiangsu and the lowest in central Jiangsu. This result indicates that the equity of health financial resource allocation was the worst in southern Jiangsu but the best in central Jiangsu.

**Table 5:** T of health financial resources allocation in Jiangsu Province

Year	Theil index		Contribution rate of intra-region (%)		Contribution rate of inter-region (%)	
	financial subsidies	Total expenditures	financial subsidies	Total expenditures	financial subsidies	Total expenditures
2014	0.1131	0.1095	7.96	17.97	92.04	82.03
2015	0.1185	0.1102	8.31	19.60	91.69	80.40
2016	0.1351	0.1116	9.32	19.14	90.68	80.86
2017	0.1301	0.1093	7.66	18.15	92.34	81.85
2018	0.1289	0.1137	7.05	17.84	92.95	82.16

**Table 6:** Proportion of differences in contribution in the south, middle and north

Year	Financial subsidies(%)			Total expenditures(%)		
	South	Middle	North	South	Middle	North
2014	35.71	7.55	56.74	32.97	0.75	66.28
2015	39.10	9.70	51.20	31.54	0.79	67.68
2016	35.26	2.79	61.95	33.67	1.23	65.10
2017	59.45	1.97	38.58	37.94	0.88	61.19
2018	66.67	4.82	28.51	41.70	1.26	57.04

**Table 7:** T of health financial resources allocation in the south, middle, north

Year	Financial subsidies			Total expenditures		
	South	Middle	North	South	Middle	North
2014	0.0121	0.0031	0.0099	0.0245	0.0007	0.0252
2015	0.0145	0.0044	0.0097	0.0257	0.0008	0.0282
2016	0.0167	0.0016	0.0151	0.0271	0.0012	0.0268
2017	0.0223	0.0009	0.0074	0.0284	0.0008	0.0234
2018	0.0228	0.0020	0.0050	0.0319	0.0012	0.0223

Figure 3 exhibits the HRDI of health financial resources. The results show that the index of financial subsidy income and total expenditure density in Jiangsu Province has been gradually improving. The HRDI in southern Jiangsu was the highest and the fastest growing, while that in northern Jiangsu was the lowest and slowest growing (Fig. 3g, i). Based on the further analysis of 13 cities under the jurisdiction of Jiangsu Province (Fig. 3h, j), the HRDI of 5 cities in southern Jiangsu is at the forefront. In particular, the HRDI of Nanjing was much higher than the provincial average and that of other cities, while the 5 cities in northern Jiangsu were at the bottom. This result indicates that there are great differences among regions in Jiangsu Province.

### The efficiency of health resource allocation in Jiangsu Province

The indicators of the input variables show an upward trend in Table 8, and the number of outpatient visits and total income in output variables also increased every year, but the hospitalization rate of the whole province decreased in 2018. Table 9 shows that the average value of TE was above 0.93, and the overall technical efficiency was high. However, PTE has gradually declined since 2016, limiting the improvement in TE. The minimum values of TE and SE are only approximately 0.65, and there is a waste of resources in some regions. According to the simulation data for 2019 to reflect the variables that need to be adjusted for each city, it can be clearly seen that Wuxi and Lianyungang need to make corresponding adjustments (Table 10).

**Table 8:** Descriptive statistics of inputs and outputs in Jiangsu Province

Year	Items	input					output		
		I_1_	I_2_	I_3_	I_4_	I_5_	O_1_	O_2_	O_3_
2014	Mean	0.407	4.831	7.312	24.515	226.203	4051	3.33	1,525,451.2
	Maxi	0.607	5.384	9.181	44.277	442.172	8597	4.98	3,656,080.0
	Mini	0.252	4.057	6.454	9.107	120.630	2284	1.84	625,023.3
2015	Mean	0.405	5.072	7.648	30.838	254.991	4202	3.38	1,705,738.8
	Maxi	0.605	5.739	9.582	60.050	505.060	9132	5.07	4,206,975.1
	Mini	0.254	4.255	6.721	11.128	134.955	2399	1.82	696,777.0
2016	Mean	0.407	5.414	8.043	36.034	284.206	4248	3.55	1,894,825.4
	Maxi	0.606	6.085	10.44	72.121	574.855	9328	5.09	4,779,372.6
	Mini	0.269	4.585	6.875	10.505	153.023	2347	1.98	773,769.8
2017	Mean	0.404	5.719	8.452	38.339	312.784	4495	3.60	2,082,306.0
	Maxi	0.598	6.592	11.238	82.821	643.702	9696	5.09	5,399,833.1
	Mini	0.281	4.761	7.225	14.822	174.079	2437	2.03	853,309.7
2018	Mean	0.416	5.974	8.936	44.035	345.758	4572	3.59	2,314,235.0
	Maxi	0.597	7.144	12.294	95.946	746.919	9907	4.80	6,259,733.8
	Mini	0.296	4.888	7.403	18.096	192.053	2446	2.00	936,204.9

I_1_: institutions (/1000 person), I_2_: beds (/1000 person), I_3_: health workers (/1000 person), I_4_: government financial subsidies (billion RMB), I_5_: total expenditures (billion RMB), O_1_: outpatient volume (ten thousand people), O_2_: annual hospitalization rate (%), O_3_: general incomes (billion RMB)

**Table 9:** TE and SE of health resource allocation in Jiangsu Province

Year	TE			PTE			SE		
	Mean	Maxi	Mini	Mean	Maxi	Mini	Mean	Maxi	Mini
2014	0.931	1	0.647	0.995	1	0.962	0.936	1	0.647
2015	0.931	1	0.631	0.998	1	0.978	0.932	1	0.631
2016	0.929	1	0.639	0.999	1	0.986	0.930	1	0.639
2017	0.929	1	0.643	0.998	1	0.976	0.931	1	0.643
2018	0.947	1	0.674	0.993	1	0.916	0.953	1	0.674

*TE* Overall technical efficiency, *PTE* Pure technical efficiency, *SE* Scale efficiency = TE/PTE

**Table 10:** Variation of inputs and outputs needed to be adjusted in 2019

City	input					output		
	I_1_	I_2_	I_3_	I_4_	I_5_	O_1_	O_2_	O_3_
Nanjing	0	0	0	0	0	0	0	0
Wuxi	−0.031	−1.358	0	−4.537	−77.563	511.220	0.099	0
Changzhou	0	0	0	0	0	0	0	0
Suzhou	0	0	0	0	0	0	0	0
Zhengjiang	0	0	0	0	0	0	0	0
Nantong	0	0	0	0	0	0	0	0
Yangzhou	0	0	0	0	0	0	0	0
Taizhou	0	0	0	0	0	0	0	0
Xuzhou	0	0	0	0	0	0	0	0
Lianyungang	− 0.121	− 0.071	0	− 0.282	−5.125	755.590	0.350	242,561.070
Huaian	0	0	0	0	0	0	0	0
Yancheng	0	0	0	0	0	0	0	0
Suqian	0	0	0	0	0	0	0	0

### The productivity of health resource allocation in Jiangsu Province

We use the MPI of the annual average to analyse the productivity of health resource allocation from 2012 to 2016 (Table 11). The TFPC was less than 1 from 2014 to 2018, and the lowest was 0.968 in 2017–2018, which indicates that productivity had decreased by 3.2%. A further analysis showed that the decrease in TC was the main reason for the decrease in TFPC. Although PTEC is decreasing annually, TEC is increasing due to the increase in SEC. With respect to frequency distribution, total factor changes declined from 2017 to 2018 for up to 11 cities. Table 12 shows the average MPI of each city from 2014 to 2018. The TFPC of five cities exceeded one, accounting for 38.5% of the 13 cities in Jiangsu Province, which shows that productivity improved. TEC and SEC values exceeded 1 in all cities except Wuxi. However, the TC value of 9 cities was less than 1, and the PTEC value of 2 cities was less than 1. TC reduction is the key reason for the decrease in TFPC in Jiangsu Province.

**Table 11:** MPI summary of annual means and frequency distribution by year

Year	TEC	TC	PETC	SEC	TFPC
2014–2015	0.999	0.993	1.007	0.992	0.991
2015–2016	0.998	0.994	0.990	1.008	0.992
2016–2017	1.001	0.992	0.998	1.004	0.993
2017–2018	1.02	0.949	0.988	1.033	0.968
Frequency distribution(2014–2015)					
> 1	4	7	1	4	5
1	6	0	11	6	0
< 1	3	6	1	3	8
Frequency distribution(2015–2016)					
> 1	4	5	1	5	6
1	7	1	11	7	0
< 1	2	7	1	1	7
Frequency distribution(2016–2017)					
> 1	4	7	1	4	8
1	6	0	11	6	0
< 1	3	6	1	3	5
Frequency distribution(2017–2018)					
> 1	6	1	0	7	2
1	6	0	11	6	0
< 1	1	12	2	0	11

*TEC* Technical efficiency changes, *TC* Technological changes, *PTEC* Pure technical efficiency changes, *SEC* Scale efficiency changes, *TFPC* Total factor productivity changes; A score > 1 indicates growth; a score of 1 signifies stagnation; a score < 1 indicates decline or deterioration

**Table 12:** MPI summary of means by city

	TEC	TC	PETC	SEC	TFPC
Nanjing	1	1.058	1	1	1.058
Wuxi	0.958	1.006	0.959	0.998	0.964
Changzhou	1	0.997	1	1	0.997
Suzhou	1	0.979	1	1	0.979
Zhengjiang	1.01	1	1	1.010	1.010
Nantong	1	0.992	1	1	0.992
Yangzhou	1.047	0.991	1	1.047	1.038
Taizhou	1.024	1	1	1.024	1.023
Xuzhou	1	0.967	1	1	0.967
Lianyungang	1.001	0.930	0.974	1.028	0.931
Huaian	1.011	1.009	1.010	1.001	1.021
Yancheng	1.008	0.940	1	1.008	0.948
Suqian	1	0.901	1	1	0.901
Mean	1.004	0.982	0.996	1.009	0.986

## Discussion

After years of reform and development, medical and health services in Jiangsu Province have made great progress. The results of this study corroborated that all resources—including the total number of medical institutions, beds, and health workers; the numbers of each resource per thousand people and per square kilometre; and medical financial subsidies and total expenditures—showed an upward trend in Jiangsu Province. However, in terms of the distribution of medical institutions at all levels, the number of beds and health workers was the largest in hospitals; additionally, the growth rate of these resources was the fastest, far higher than that of primary medical institutions. This finding shows that although Jiangsu Province has issued a series of policies to support primary medical institutions in recent years, there is still a trend of the accelerated expansion of large hospitals. It is necessary to gradually pass the excessive medical resources of large hospitals to grassroots institutions and communities. According to the distribution of financial resources in different regions, there are great regional differences in these resources in Jiangsu Province. The excessive concentration of financial resources is manifested in the rich medical resources and high levels of medical care in some cities, which is not conducive to the improvement of the overall medical care level of Jiangsu Province. In the future, the government should clearly address the problem of health resource allocation and strive to narrow the gap in medical resource allocation in different regions [23].

This study comprehensively analysed the equity and efficiency of health care resource allocation in Jiangsu Province in recent years. The distribution of health care resources in Jiangsu Province is generally fair: the fairness distribution according to the population is higher than that according to the geographical area, and the distribution of primary health resources is better than that of financial resources. This finding is consistent with the results of other studies on the equity of health resource allocation in China [24, 25]. There are two main reasons for this result: on the one hand, the policies issued by the government aim mainly to meet the medical needs of the population, not the needs of the regional layout [26]; on the other hand, there is a gap in the economic development of Jiangsu Province, which has a certain impact on the supply of medical resources [16]. The results for T show that the unfair allocation of resources in Jiangsu Province was caused mainly by differences among regions. The fairness of health resource allocation in the middle of the province was better than that in southern and northern Jiangsu. According to the HRDI, there are both excesses and deficiencies in medical resources. There is a surplus of medical institutions in northern Jiangsu, but the number of beds and medical staff is insufficient, while in southern Jiangsu, the opposite is true. Xu also verified this result in southern cities in Jiangsu, where more health workers and material resources are available, while northern areas have fewer resources [8]. This finding shows that southern Jiangsu needs to transfer excess high-quality human and financial resources to the northern and the central regions of Jiangsu. In addition, population and geographical factors should be taken into account to allocate health resources scientifically and reasonably [27, 28].

The analysis of efficiency and productivity confirmed that the overall efficiency of health resource allocation in Jiangsu Province was high, but TFPC decreased annually. The mean scores of TE, PTE and SE were higher than 0.92. The mean TE scores for Jiangsu Province are still higher than those for the Spanish region of Extremadura (0.833) [29], South Korea (0.886), Oman (0.692) and all of China (0.806) [30]. The results for medical resource allocation in most cities of Jiangsu Province were satisfactory, but it cannot be ignored that the minimum TE and SE of 13 cities were only approximately 0.65, which means there was a waste of resources in some cities. Based on the current distribution of medical resources, Jiangsu Province needs to further optimize the allocation of medical resources and focus on key cities, such as Wuxi and Lianyungang, where the DEA results were invalid, to improve efficiency. It is necessary to reduce input and increase output over time. Using the Malmquist index to study dynamic efficiency, the results show that the TFPC was less than 1 from 2014 to 2018. In terms of the specific situation in each city, the TFPC of 11 cities in Jiangsu Province has declined in the past 5 years. Because TFPC can be decomposed into TEC and TC, TE changed positively from 2016 to 2018, and the decrease in TC was the key reason for the decline in TFPC. Li et al. asserted that technological progress will contribute to the development of health care services [18]. Therefore, to improve the efficiency of health care resources and ensure the sustainable development of medical services, Jiangsu Province and its municipalities must pay attention to technological progress and improve technological efficiency. In addition, it is necessary to improve the service ability of basic medical institutions [31], strengthen the cooperation among medical institutions at all levels [32], and further improve training and incentive mechanisms for doctors [33].

## Limitations

Although we use a variety of methods to evaluate the equity and efficiency of health care resource allocation in Jiangsu Province, there are still some limitations to this study. First, although the selection of indicators is commonly used in similar research and indicators for health workers are included, we did not further subdivide these workers into doctors, nurses and other personnel. Second, this study mainly analyses the fairness of health care resource allocation from the perspective of population and geography, without considering the actual health status and health service needs of different regions. Third, due to the limitations of the basic DEA method, there are no bias adjustments for efficiency and productivity scores.

## Conclusion

In the past 5 years, the total amount of health resources in Jiangsu Province has increased steadily, and the overall equity of health care resource allocation has been good. The equity of basic medical resources was better than that of financial resources, and the equity of geographical allocation was better than that of population allocation. However, there were great differences among regions: health care resources in southern Jiangsu were plentiful, while those in central and northern Jiangsu were relatively lacking. The overall efficiency of health care resource allocation was high, but some cities faced low efficiency. In addition, the total factor productivity of the whole province has declined. Therefore, to ensure the sustainable development of medical services, the government should further optimize the allocation of health resources, comprehensively consider geographical and demographic factors, and strive to narrow the gap in medical resources in different regions. In addition, to increase the utilization efficiency of limited medical resources, technical efficiency needs to be improved by developing the service ability of basic medical institutions, strengthening cooperation among medical institutions at all levels, and improving training and incentive mechanisms for doctors.

## Supplementary Information

Additional file 1: **Fig. S1.**The Lorenz curves of health care resources in Jiangsu Province from 2015 to 2017. **A-F** show the Lorenz curves of primary resources allocated by geographical area (**A-C**) and population (**D-F**). **G-L** show the Lorenz curves of financial resources allocated by geographical area (**G-I**) and population (**J-L**). **A, D, G, J** are in 2015; **B, E, H, K** are in 2016; **C, F, I, L** are in 2017.

## Data Availability

Please contact author for data requests.

## Abbreviations

DEA: Data envelopment analysis
G: Gini coefficient
HRDI: Health resource density index
T: Theil index
MPI: Malmquist productivity index
TE: Technical efficiency
PTE: Pure technical efficiency
SE: Scale efficiency
TEC: Technical efficiency changes
TC: Technological changes
PTEC: Pure technical efficiency changes
SEC: Scale efficiency changes
TFPC: Total factor productivity changes
