# Supplementary figures and images for: Equity and efficiency of health care resource allocation in Jiangsu Province, China
Source: Int J Equity Health. 2020 Nov 27;19:211. doi: 10.1186/s12939-020-01320-2 (PMC7694921; doi:10.1186/s12939-020-01320-2)

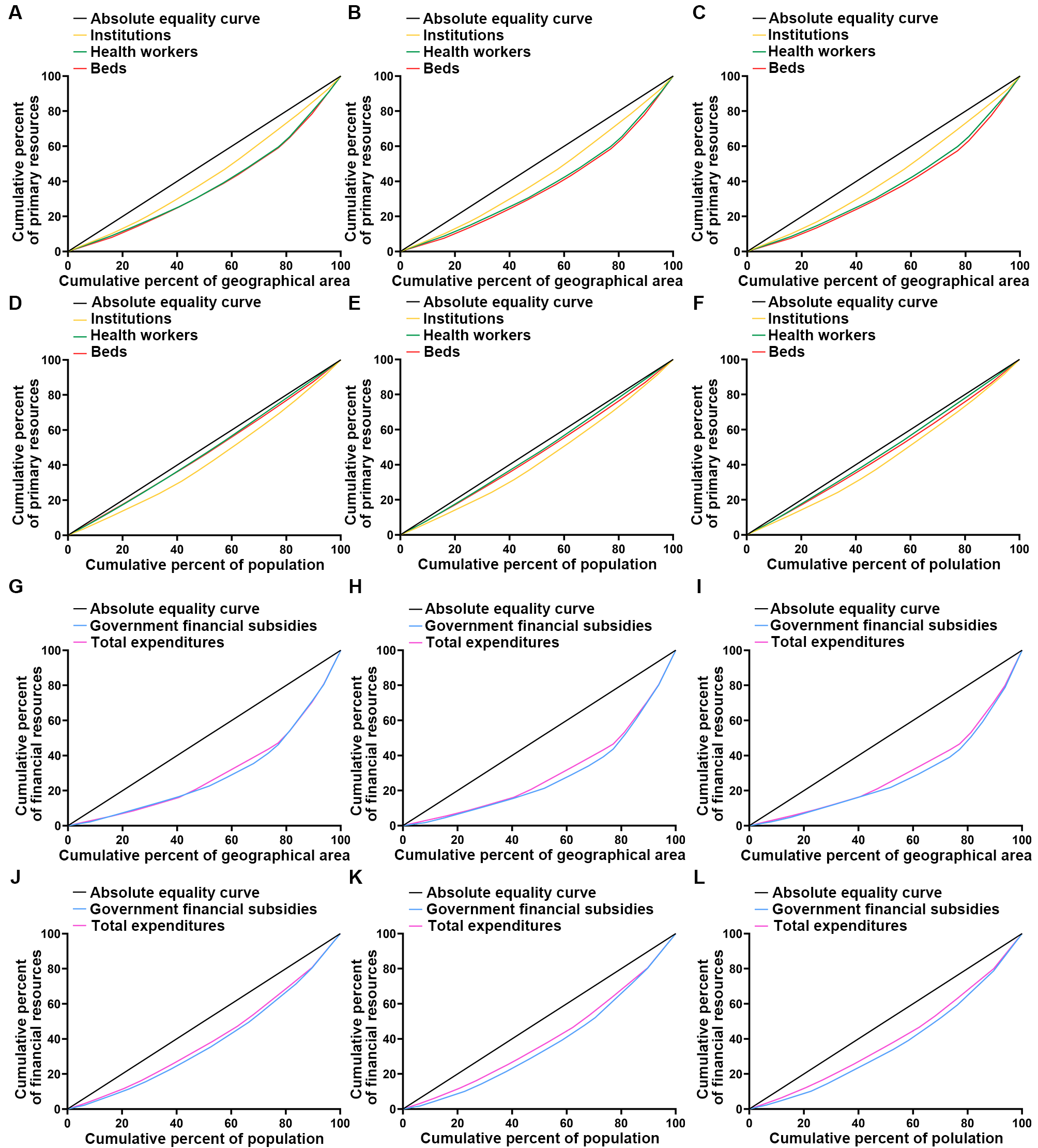

Supplement: Supplementary file 1 — Fig. S1.The Lorenz curves of health care resources in Jiangsu Province from 2015 to 2017. A-F show the Lorenz curves of primary resources allocated by geographical area (A-C) and population (D-F). G-L show the Lorenz curves of financial resources allocated by geographical area (G-I) and population (J-L). A, D, G, J are in 2015; B, E, H, K are in 2016; C, F, I, L are in 2017. [file 12939_2020_1320_MOESM1_ESM.tif]
